# Two hypotheses of dense breasts and viral infection for explaining incidence of breast cancer by age group in Korean women

**DOI:** 10.4178/epih/e2014020

**Published:** 2014-09-26

**Authors:** Jong-Myon Bae

**Affiliations:** Department of Preventive Medicine, Jeju National University School of Medicine, Jeju, Korea

**Keywords:** Breast neoplasm, Risk factors, Cancer screening, Mammography, Papillomaviridae

## Abstract

Breast cancer, the second leading type of cancer in Korean women, has shown increasing incidence over the past 10 years. However, the curves of incidence by age group cast doubt on the birth cohort effect hypothesis. To explain the curves, here I suggest two alternative hypotheses of breast density and viral infection based on pre-existing evidences. Evaluating these hypotheses would require important clues to find unknown risk factors of breast cancer and to plan more effective strategies for breast cancer control in Korean women.

## INTRODUCTION

Breast cancer ranks first in incidence and mortality rates for women throughout the world [1]. The number of patients with breast cancer in developing countries has recently become comparable to that of women in developed countries [2]; in Asia in particular, the incidence is increasing in younger age groups [3,4]. Similarly, the incidence of breast cancer in South Korea has been increasing over the last 10 years [5], which is being interpreted as a birth cohort effect [3,6] from a westernized lifestyle [7-9] or the effects of early screening program for breast cancer [5].

**Figure 1.**
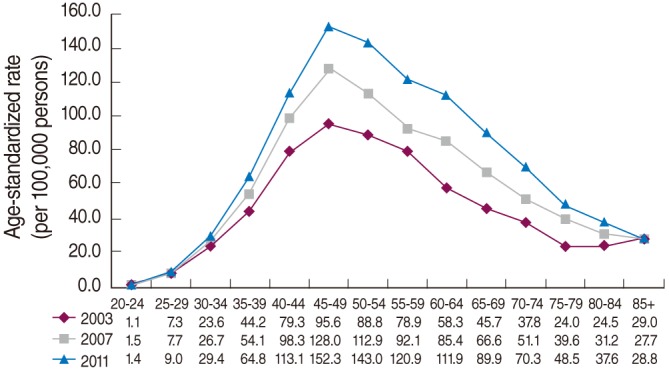
Curves of breast cancer incidence in Korean women by age group and in 4-year intervals.

However, when incidences are examined by age group, an unusual phenomenon occurs since, as seen in Figure 2 of Bae [10], the peak appears in the age range of 45-49 years and declines thereafter. Moreover, Figure 1(b) of Lee et al. [11] shows that the incidence curves for the years 1993-2002 appear the same. Figure 1 in this paper shows that the incidence curves by age group were redrawn for different years. This appearance of peaking at 45-49 years, declining thereafter, and showing no changes for the past 20 years can be interpreted as no or little cohort effect in the three observation axes in the age-period-cohort analysis [12].

As such, it can be suspected that the characteristics of breast cancer incidence in Korean women could involve reasons other than a birth cohort effect from lifestyle westernization. In an effort to explain these reasons, an opportunity to identify the causal factors specific for Korean women is created and effective preventative measures can be established. Therefore, here I present two hypotheses based on literature reviews on breast cancer.

## THE FIRST HYPOTHESIS: CHANGES IN DENSE BREAST DISTRIBUTION BY AGE GROUP

The evidence for this hypothesis is described in detail in the forthcoming published report by Kim & Bae [13]. Summary and supplementary explanations are given here.

The identification of dense breasts on mammography increases the breast cancer occurrence rate by 4-6 times in Western Caucasian women; as such, it has become the strongest known risk factor to date [14-17]. In particular, in terms of the risk associated with dense breasts, it is reportedly increasing the incidence of breast cancer diagnoses in young women <50 years of age [18]. However, only a few studies have closely examined the hypothesis that dense breasts represent a risk factor for breast cancer in Korean women, and its effect is not yet known [19-21]. The reasons for these are twofold: first, Asian women, including Korean women, have more dense breasts than Caucasian women [22,23]; and second, although breast density decreases rapidly after the 30s due to pregnancy, childbirth, and lactation, most studies did not reflect changing trends by age group since only mammographic density immediately preceding breast cancer diagnosis was examined.

On the other hand, according to a report that risk of dense breasts is maintained even after 10 years [24], examining the previous mammographic density from 10 years prior to a cancer diagnosis is also necessary. The fact that the 35-39-year age group, which is 10 years younger than the 45-49-year age group and shows the peak in incidence, had the highest rate of dense breasts [22] leads to a stronger implication of the association [13]. In addition, over the past 10 years, the proportion of dense mammography in screening is increasing in every age group in [25]. In these respects, useful evidences can be obtained from cohort studies that track cancer incidences using multiple test results of women who have repeatedly undergone breast cancer screenings [13].

## THE SECOND HYPOTHESIS: CHANGES IN INFECTION RATES OF CANCER-INDUCING VIRUSES

As breast cancer in developing countries is becoming more common in relatively younger age groups, the possibility of infection is being actively suggested, leading to the emergence of the concept of breast cancer being an infectious disease [26,27]. With Korean women also showing the highest incidence at ages 45-49 years [5,10], we must consider the possible association with infection. To date, three types of viruses, mouse mammary tumor virus (MMTV), Epstein-Barr virus (EBV), and human papillomavirus (HPV), have been reported to increase the risk of breast cancer occurrence [28].

### Mouse mammary tumor virus

MMTV was isolated from mice with breast cancer that were raised in the same cage, so it is natural to suspect that MMTV can also cause breast cancer in humans [29]. The work by Labat [30] details the laboratory experiment results on the association between MMTV and breast cancer. In 2012, Glenn et al. [31] claimed that co-infections with the following two viruses increased the risk of breast cancer compared to that of MMTV alone.

### Epstein-Barr virus

EBV, widely known to cause Burkitt’s lymphoma [32], received much attention after Labrecque et al. [33] reported that EBV-related DNA were found in the tissues and blood of patients with breast cancer. However, other research papers have reported no association [34-36], causing much confusion. Glaser et al. [37] and Lawson et al. [38] concluded that additional studies are needed through their review of articles on related studies.

### Human papillomavirus

HPV, known as a virulence factor for uterine cervical cancer [39], reportedly has a relationship with breast cancer [40-42], and the systematic reviews revealed an association [43,44]. Li et al. [44] reported that 24.49% of breast cancer cases were related to HPV; geographically, this finding was more common in Asians (32.42%) than in Europeans (12.91%). However, a search for studies on Korean women returned only one report indicating that HPV DNA was detected in breast cancer tissue by a DNA chip [45].

The mechanism by which HPV infection causes breast cancer is explained by HPV infiltration through the mammalian duct from the genital to oral route of the sex partner or propagation through the blood [27,46]. Moreover, HPV-related breast cancer is known to occur in younger age groups [31,47], with higher malignancy [48,49]. Furthermore, while studies from Europe and Iran have reported an association between HPV types 16 and 18 and breast cancer [41,50], another study reported that HPV type 33 was related to breast cancer in Japanese and Chinese women [51]. However, as shown in Table 1, most reports on breast cancer tissues in Asians have shown negative results[52-54]. As some studies claim that the reason why existing studies failed to identify the relationship with HPV was due to the examined breast cancer tissues being treated with paraffin [55] or limitations of the experimental techniques [42].

## CONCLUSIONS AND RECOMMENDATIONS

In this paper, two hypotheses that were proposed to explain the trends in increasing incidence of breast cancer in Korean women for the last 20 years were investigated. To obtain evidence that support the first hypothesis that dense breasts are related to breast cancer occurrence, the direction of future studies should be to: (1) verify whether the distribution of dense breasts by age correlates with the annual incidence of breast cancer; (2) use a case-control study method to identify the association between dense breasts and well-known breast cancer risk or protective factors; and (3) conduct a cohort study to verify the relative risk for the onset of breast cancer based on patterns of change in breast density in subsequent screening mammography. If these studies can prove the hypotheses, it would be necessary to establish a new screening strategy, in which patients with dense breasts are selected as a risk group; breast ultrasonography, in addition to mammography, is performed regularly; and the interval of follow-ups is better coordinated [56]. To obtain evidences for the second hypothesis that HPV is related to breast cancer in Korean women, the direction of future studies should be to (1) obtain fresh breast cancer tissues, if possible, to determine the degree of association of HPV DNA to breast cancer using a case-control study, and (2) if relevance is found, identify what subtype is responsible. Moreover, the proposed epidemiological study directions are to: (3) investigate whether patients with HPV infection detected by a Pap smear have higher future risks of breast cancer, and (4) identify any difference in breast cancer occurrence based on HPV vaccination. If a relationship between HPV and breast cancer risk can be established through these studies, HPV vaccinations for the prevention of cervical cancer can also lead to the prevention of breast cancer [40,57-59]. Under the current circumstances in South Korea, co-inoculation of HPV vaccine during the time of rubella vaccination for reproductive-age women should be considered. In conclusion, it is hoped that this paper will stimulate active epidemiological and experimental studies in Korean women.

## Figures and Tables

**Table 1.** Studies on Asians that evaluated the association between human papillomavirus infection and female breast cancer risk

Design	Likely	Unlikely
Cancer tissues only	[51] (Japan & China)	[53] (Japan)
		[54] (India)
Case-control comparison	[50] (Iran)	[52] (Taiwan)

Values are presented as [reference number] (nation of subjects).

## References

[1] Jemal A, Bray F, Center MM, Ferlay J, Ward E, Forman D (2011). Global cancer statistics. CA Cancer J Clin.

[2] Ferlay J, Shin HR, Bray F, Forman D, Mathers C, Parkin DM (2010). Estimates of worldwide burden of cancer in 2008: GLOBOCAN 2008. Int J Cancer.

[3] Shin HR, Joubert C, Boniol M, Hery C, Ahn SH, Won YJ (2010). Recent trends and patterns in breast cancer incidence among Eastern and Southeastern Asian women. Cancer Causes Control.

[4] Keramatinia A, Mousavi-Jarrahi SH, Hiteh M, Mosavi-Jarrahi A (2014). Trends in incidence of breast cancer among women under 40 in Asia. Asian Pac J Cancer Prev.

[5] Jung KW, Won YJ, Kong HJ, Oh CM, Lee DH, Lee JS (2014). Cancer statistics in Korea: incidence, mortality, survival, and prevalence in 2011. Cancer Res Treat.

[6] Shen YC, Chang CJ, Hsu C, Cheng CC, Chiu CF, Cheng AL (2005). Significant difference in the trends of female breast cancer incidence between Taiwanese and Caucasian Americans: implications from age-period-cohort analysis. Cancer Epidemiol Biomarkers Prev.

[7] Moore MA (2014). Cancer control programs in East Asia: evidence from the international literature. J Prev Med Public Health.

[8] Bray F, McCarron P, Parkin DM (2004). The changing global patterns of female breast cancer incidence and mortality. Breast Cancer Res.

[9] Youlden DR, Cramb SM, Dunn NA, Muller JM, Pyke CM, Baade PD (2012). The descriptive epidemiology of female breast cancer: an international comparison of screening, incidence, survival and mortality. Cancer Epidemiol.

[10] Bae JM (2014). On the benefits and harms of mammography for breast cancer screening in Korean women. Korean J Fam Pract.

[11] Lee JH, Yim SH, Won YJ, Jung KW, Son BH, Lee HD (2007). Population-based breast cancer statistics in Korea during 1993-2002: incidence, mortality, and survival. J Korean Med Sci.

[12] Gardner MJ, Osmond C (1983). Interpretation of disease time trends: is cancer on the increase? A simple cohort technique and its relationship to more advanced models. J Epidemiol Community Health.

[13] Kim EH, Bae JM (2014). Potential availability of dense mammogrphy for prevention of breast cancer in Korean women. Korean J Fam Pract.

[14] Boyd NF, Lockwood GA, Byng JW, Tritchler DL, Yaffe MJ (1998). Mammographic densities and breast cancer risk. Cancer Epidemiol Biomarkers Prev.

[15] Boyd NF, Guo H, Martin LJ, Sun L, Stone J, Fishell E (2007). Mammographic density and the risk and detection of breast cancer. N Engl J Med.

[16] Boyd NF, Rommens JM, Vogt K, Lee V, Hopper JL, Yaffe MJ (2005). Mammographic breast density as an intermediate phenotype for breast cancer. Lancet Oncol.

[17] Veronesi U, Boyle P, Goldhirsch A, Orecchia R, Viale G (2005). Breast cancer. Lancet.

[18] Bertrand KA, Tamimi RM, Scott CG, Jensen MR, Pankratz V, Visscher D (2013). Mammographic density and risk of breast cancer by age and tumor characteristics. Breast Cancer Res.

[19] Cho JJ, Song HJ, Koh EY, Song YM, Han BK, Yun YS (2006). Mammographic breast density and risk factors of breast cancer in Korean women using multicenter study. J Korean Acad Fam Med.

[20] Kang EY, Shin JH, Kang SG, Hwang YN, Cha ES, Song SW (2007). Relationship between mammographic dense breast and other risk factors of breast cancer in Korean women. Korean J Fam Med.

[21] Park IH, Ko K, Joo J, Park B, Jung SY, Lee S (2014). High volumetric breast density predicts risk for breast cancer in postmenopausal, but not premenopausal, Korean women. Ann Surg Oncol.

[22] Kim SH, Kim MH, Oh KK (2000). Analysis and Comparison of Breast Density according to Age on Mammogram between Korean and Western women. J Korean Radiol Soc.

[23] El-Bastawissi AY, White E, Mandelson MT, Taplin S (2001). Variation in mammographic breast density by race. Ann Epidemiol.

[24] Yaghjyan L, Colditz GA, Rosner B, Tamimi RM (2013). Mammographic breast density and subsequent risk of breast cancer in postmenopausal women according to the time since the mammogram. Cancer Epidemiol Biomarkers Prev.

[25] Kim EH, Bae JM (2014). Potential availability of dense mammography for prevention of breast cancer in Korean women. Korean J Fam Pract.

[26] Lawson JS, Glenn WK, Whitaker NJ (2010). Breast cancer as an infectious disease. Womens Health (Lond Engl).

[27] Lawson JS, Kan CY, Iacopetta BJ, Whitaker NJ (2006). Are some breast cancers sexually transmitted?. Br J Cancer.

[28] Amarante MK, Watanabe MA (2009). The possible involvement of virus in breast cancer. J Cancer Res Clin Oncol.

[29] Mesa-Tejada R, Keydar I, Ramanarayanan M, Ohno T, Fenoglio C, Spiegelman S (1978). Detection in human breast carcinomas of an antigen immunologically related to a group-specific antigen of mouse mammary tumor virus. Proc Natl Acad Sci U S A.

[30] Labat ML (1998). Possible retroviral etiology of human breast cancer. Biomed Pharmacother.

[31] Glenn WK, Heng B, Delprado W, Iacopetta B, Whitaker NJ, Lawson JS (2012). Epstein-Barr virus, human papillomavirus and mouse mammary tumour virus as multiple viruses in breast cancer. PLoS One.

[32] Orem J, Mbidde EK, Lambert B, de Sanjose S, Weiderpass E (2007). Burkitt’s lymphoma in Africa, a review of the epidemiology and etiology. Afr Health Sci.

[33] Labrecque LG, Barnes DM, Fentiman IS, Griffin BE (1995). Epstein-Barr virus in epithelial cell tumors: a breast cancer study. Cancer Res.

[34] Chu JS, Chen CC, Chang KJ (1998). In situ detection of Epstein-Barr virus in breast cancer. Cancer Lett.

[35] Chu PG, Chang KL, Chen YY, Chen WG, Weiss LM (2001). No significant association of Epstein-Barr virus infection with invasive breast carcinoma. Am J Pathol.

[36] Speck P, Callen DF, Longnecker R (2003). Absence of the Epstein-Barr virus genome in breast cancer-derived cell lines. J Natl Cancer Inst.

[37] Glaser SL, Hsu JL, Gulley ML (2004). Epstein-Barr virus and breast cancer: state of the evidence for viral carcinogenesis. Cancer Epidemiol Biomarkers Prev.

[38] Lawson JS, Günzburg WH, Whitaker NJ (2006). Viruses and human breast cancer. Future Microbiol.

[39] Cox JT (2006). The development of cervical cancer and its precursors: what is the role of human papillomavirus infection?. Curr Opin Obstet Gynecol.

[40] Cuzick J (2010). Long-term follow-up in cancer prevention trials (It ain’t over ‘til it’s over). Cancer Prev Res (Phila).

[41] Damin AP, Karam R, Zettler CG, Caleffi M, Alexandre CO (2004). Evidence for an association of human papillomavirus and breast carcinomas. Breast Cancer Res Treat.

[42] Wang T, Chang P, Wang L, Yao Q, Guo W, Chen J (2012). The role of human papillomavirus infection in breast cancer. Med Oncol.

[43] Simões PW, Medeiros LR, Simões Pires PD, Edelweiss MI, Rosa DD, Silva FR (2012). Prevalence of human papillomavirus in breast cancer: a systematic review. Int J Gynecol Cancer.

[44] Li N, Bi X, Zhang Y, Zhao P, Zheng T, Dai M (2011). Human papillomavirus infection and sporadic breast carcinoma risk: a meta-analysis. Breast Cancer Res Treat.

[45] Choi YL, Cho EY, Kim JH, Nam SJ, Oh YL, Song SY (2007). Detection of human papillomavirus DNA by DNA chip in breast carcinomas of Korean women. Tumour Biol.

[46] Widschwendter A, Brunhuber T, Wiedemair A, Mueller-Holzner E, Marth C (2004). Detection of human papillomavirus DNA in breast cancer of patients with cervical cancer history. J Clin Virol.

[47] Dunne EF, Unger ER, Sternberg M, McQuillan G, Swan DC, Patel SS (2007). Prevalence of HPV infection among females in the United States. JAMA.

[48] Polyak K (2001). On the birth of breast cancer. Biochim Biophys Acta.

[49] Hennig EM, Suo Z, Thoresen S, Holm R, Kvinnsland S, Nesland JM (1999). Human papillomavirus 16 in breast cancer of women treated for high grade cervical intraepithelial neoplasia (CIN III). Breast Cancer Res Treat.

[50] Sigaroodi A, Nadji SA, Naghshvar F, Nategh R, Emami H, Velayati AA (2012). Human papillomavirus is associated with breast cancer in the north part of Iran. ScientificWorldJournal.

[51] Yu Y, Morimoto T, Sasa M, Okazaki K, Harada Y, Fujiwara T (1999). HPV33 DNA in premalignant and malignant breast lesions in Chinese and Japanese populations. Anticancer Res.

[52] Tsai JH, Tsai CH, Cheng MH, Lin SJ, Xu FL, Yang CC (2005). Association of viral factors with non-familial breast cancer in Taiwan by comparison with non-cancerous, fibroadenoma, and thyroid tumor tissues. J Med Virol.

[53] Khan NA, Castillo A, Koriyama C, Kijima Y, Umekita Y, Ohi Y (2008). Human papillomavirus detected in female breast carcinomas in Japan. Br J Cancer.

[54] Hedau S, Kumar U, Hussain S, Shukla S, Pande S, Jain N (2011). Breast cancer and human papillomavirus infection: no evidence of HPV etiology of breast cancer in Indian women. BMC Cancer.

[55] Antonsson A, Spurr TP, Chen AC, Francis GD, McMillan NA, Saunders NA (2011). High prevalence of human papillomaviruses in fresh frozen breast cancer samples. J Med Virol.

[56] Wang FL, Chen F, Yin H, Xu N, Wu XX, Ma JJ (2013). Effects of age, breast density and volume on breast cancer diagnosis: a retrospective comparison of sensitivity of mammography and ultrasonography in China’s rural areas. Asian Pac J Cancer Prev.

[57] Rambout L, Hopkins L, Hutton B, Fergusson D (2007). Prophylactic vaccination against human papillomavirus infection and disease in women: a systematic review of randomized controlled trials. CMAJ.

[58] Harputluoglu H, Dizdar O, Altundag K (2006). Prophylactic human papilloma virus vaccines for cervical cancer may also prevent development of breast and oropharyngeal cancers in women. Med Hypotheses.

[59] Mayeaux EJ (2006). Harnessing the power of prevention: human papillomavirus vaccines. Curr Opin Obstet Gynecol.

