# Supplementary material for: Two hypotheses of dense breasts and viral infection for explaining incidence of breast cancer by age group in Korean women
Source: Epidemiol Health. 2014 Sep 26;36:e2014020. doi: 10.4178/epih/e2014020 (PMC4220604; doi:10.4178/epih/e2014020)
Supplement: Supplementary file 1 [file epih-36-e2014020-supplementary.pdf]

한국 여성유방암의 연령별 발생 추이에 대한 두 가설

Two Hypotheses of Dense Breasts and Viral Infection for explaining the incidence rates of breast cancer by age group in Korean women

Abstract

Breast cancer is the second leading type of cancers in Korean women, and showed increasing incidence rates over 10 years. But the curves of incidence rates by age group cast doubt on the birth cohort effect hypothesis. To explain the curves, this author is suggesting two other hypotheses such as breast density and viral infection. These are proposed based on pre-existing evidences. Trying to evaluate the hypotheses would take the important clues to find unknown risk factors of breast cancer and to plan more effective strategies for breast cancer control in Korean women.

Key words: Breast neoplasm, Risk factors, Cancer Screening, Mammography, Papillomaviridae

1. 서론

유방암은 전세계적으로 여성 암의 발생 및 사망 1위를 차지하고 있다 [1]. 최근에는 개발도상국과 선진국의 환자 수가 유사하며 [2], 특히 아시아권에서의 젊은 연령층의 발생률이 증가하고 있다 [3, 4]. 한국도 지난 10년간 유방암 발생률은 증가하고 있는 추세이며 [5], 이에 대하여는 서구화된 생활습관[6-8]에 따른 코호트 효과 (cohort effect) [3, 9]와 유방암 조기검진의 효과로 해석하고 있다 [5].

그런데, 전체적으로 증가한 발생률을 연령군별로 살펴보면 특이한 현상을 볼 수 있다. Bae [10]에 제시된 Fig. 2처럼 45-49세를 정점을 이룬 다음 이후 연령대에서 떨어지는 모습은 그대로 유지한 채 봉우리 높이만 치솟는 모습을 보이고 있기 때문이다. 또한 Shin et al. [11]에 제시된 Fig. 1(b)를 보면 1993-2002년의 발생률 곡선에서도 여전히 같은 모습을 보여주고 있다. 본 원고의 Fig. 1은 연도를 달리하여 연령별 발생률 곡선을 다시 그려본 것이다. 이렇게 45-49세를 정점으로 이후 연령대에서 감소하는 모습을 지난 20여년 간 변함없이 보여준다는 것은 연령-기간-코호트 (age-period-cohort analysis)의 3가지 관찰 축에서 코호트 효과는 없거나 미미하다고 해석할 수 있겠다 [12].

이렇듯 한국 여성 유방암의 발생률 특성은 서구화에 따른 코호트 효과가 아닌 다른 이유가 개입되어 있을 것으로 의심할 수 있다. 이를 설명하려는 노력에서 한국 여성의 특별한 발생 요인을 찾을 기회를 가지고, 효과적인 예방대책을 수립할 수 있을 것이다. 저자는 이런 점에서 유방암 발생에 관한 문헌 고찰을 통해 다음 2 가지 가설을 제시하고자 한다.

2. 제 1 가설: 연령별 치밀유방 분포의 변화

본 가설을 제시하게 된 근거들에 대하여는 곧 게재될 Kim & Bae[13]에서 자세히 밝혔다. 여기에서는 요약 및 보충하여 설명한다.

서구 백인여성에서 유방촬영술로 확인된 치밀유방 (mammographic density)은 유방암 발생을 4-6배 증가시키는 것으로 알려지면서, 현재까지 알려진 가장 강력한 위험요인으로 알려져 있다 [14-17]. 특히 치밀유방의 위험도는 50세 미만의 젊은 여성의 유방암 발생을 높이는 것으로 보고되고 있다 [18].

그런데 지금껏 한국 여성에 있어 치밀유방이 유방암의 위험요인이 된다는 가설을 규명한 연구는 소수일 뿐만 아니라 관련성을 제대로 알아내지 못하였다 [19-21]. 이런 이유로는 첫째, 한국여성을 포함한 아시아 여성은 서구백인여성에 비하여 치밀유방을 더 많이 갖고 있기 때문이다 [22, 23]. 둘째, 30대를 지나면서 임신, 분만, 수유를 하면서 치밀유방은 급격히 감소하는 과정을 밟는데, 대부분의 연구는 유방암 진단 직전의 유방촬영술의 치밀도를 살펴보면서 연령별 변화 양상을 제대로 반영하지 못하였다.

한편, 치밀유방의 위험도는 10년이 지나도 유지된다는 보고[24]에 따르면, 암 진단 이전 10년 전의 유방밀도를 살펴보는 것도 필요할 것이다. 발생률의 정점을 보이는 45-49세 연령군에서 10년 이전 연령군인 35-39세는 치밀유방을 가진 분율이 가장 높다는 사실[22]에서 더욱 관련성이 의심된다 [13]. 또한 지난 10년을 지나면서 한국여성의 암검진용 유방촬영술에 있어 치밀유방으로 진단된 경우가 연령별로 더 많아진 것으로 보고되었다 [25]. 이런 점들을 감안할 때, 유방암 조기검진자의 반복 검사 결과들을 활용하여 암발생을 추적

하는 코호트 연구를 수행한다면 유용한 근거들을 확보할 것이다 [13].

3. 제 2 가설: 암유발 바이러스 감염률 변화

개발도상국에서 유방암 발생은 상대적으로 젊은 연령층에서 이루어진다는 점에서 감염의 가능성이 적극 제기되면서 유방암은 감염병이라는 개념이 등장하게 되었다 [26, 27]. 우리나라 여성도 45-49세에 발생률이 제일 높다는 점[5, 10]에서 감염과의 관련성을 의심해 볼 필요가 있다. 현재까지 유방암 발생의 위험을 높이는 바이러스로 mouse mammary tumor virus (MMHV), Epstein-Barr virus (EBV), human papillomavirus (HPV)의 3가지가 보고되고 있다 [28].

가. MMHV

같은 우리에 사육한 쥐들에서 유방암이 발생한다는 사실에 따라 분리된 MMHV는 인간 유방에도 암을 일으킬 수 있을 것이란 의심을 하는 것은 당연할 것이다 [29]. MMHV와 유방암과의 관련한 실험실 연구결과들은 Labat의 논문 [30]을 참조하기 바란다. 2012년도 Glenn et al. [31]은 MMHV 단독보다는 아래 두 가지 바이러스와의 공통 감염이 유방암 발생 위험을 높인다고 주장하였다.

나. EBV

Burkitt's lymphoma를 일으키는 바이러스로 잘 알려진 EBV [32]는 Labrecque et al. [33]이 유방암 환자의 조직과 혈액에서 EBV 관련 DNA가 발견된다는 보고를 하면서 많은 관심을 갖게 되었다. 그러나 관련이 없다는 연구 보고들 [34-36] 도 보고되면서 매우 혼란한 가운데,

Glaser et al. [37] 과 Lawson et al. [38] 은 관련 연구들을 종설로 정리하면서 더 많은 연구가 추가로 필요하다고 결론내고 있다.

다. HPV

자궁경부암의 발병인자로 알려진 HPV [39]가 유방암에도 관련이 있다는 연구들에 대하여는 종설로 보고되었다 [40-42]. 또한 체계적 고찰 (systematic review)을 적용하여 관련성이 있다는 결론을 도출하였다 [43, 44]. 이중 Li et al. [44]은 유방암 중 HPV와 관련이 있는 경우가 24.49% 이며, 지역별로 나눌 때 유럽인은 12.91%인 반면 아시아인은 32.42%로 더 높다고 보고하였다. 그런데, 한국 여성을 대상으로 한 연구로는 DNA chip으로 유방암 조직에서 HPV DNA를 발견했다는 보고가 하나 검색될 뿐이다 [45].

HPV 감염이 유방암을 일으키는 기전은 성 파트너의 성기-구강 경로로 유두관으로 침투하거나 혈액을 통한 전파로 해석하고 있다 [27, 46]. 그리고 HPV와 관련한 유방암은 젊은 연령층에서 발생하며 [31, 47], 악성도가 더 높은 것으로 [48, 49] 알려져 있다. 또한, 유럽과 이란 연구에서는 16, 18형이 관련이 있다고 [41, 50] 발표한 반면, 일본과 중국 여성 유방암에는 33형이 관련이 있다는 보고가 있다 [51]. 그런데, Tab 1에서 보듯이 아시아인에 대한 유방암 조직에서 HPV가 관련이 없는 보고가 다수이다 [52-54]. 이렇게 기존의 연구들 중에서 HPV 관련성을 찾지 못한 연구들은 파라핀 처리를 한 유방암 조직을 사용했거나 [55], 실험기법의 한계 때문이라는 주장이 있다 [42].

4. 결어 및 제언

지난 20년간 한국 여성의 발생률 추이를 통해 그 특성을 설명하기 위해 제시한 두 가지 가설을 살펴보았다. 첫째 가설에 대하여 치밀유방이 유방암 발생과 관련이 있다는 근거들을 확보하기 위한 향후 연구 방향으로는 (1) 연령별 치밀유방의 분포가 연도별 발생률과 공조를 하는가를 확인하면서 (2) 환자-대조군 연구방법으로 치밀유방이 기존에 알려진 유방암의 위험/보호요인들과 어떤 관련을 가지는 가를 규명하고 (3) 조기암 검진자의 치밀-지방 유방의 변화양상에 따라 유방암 발생여부의 상대위험도를 코호트 연구를 수행하여 확인하는 것 등이다. 이런 연구로 가설이 입증된다면, 치밀유방 대상자는 유방암 검진에서 위험군으로 설정하고, 유방촬영술 이외 유방초음파 등을 연이어 시행하면서, 검사 간격도 더 조율하는 검진 전략을 세울 필요가 있을 것이다 [56]. 둘째 가설에 있어서 특히 HPV 관련성이 한국 여성의 유방암과 관련성이 있다는 근거들을 확보하기 위한 향후 실험실 연구 방향으로는 (1) 가급적이면 유방암 생조직 (fresh tissue)을 확보하여 환자-대조군 연구로 HPV DNA 관련성 정도를 확인하고 (2) 관련성이 있다면 어떤 아형인지를 알아낼 필요가 있다. 또한 역학 연구 방향으로는 (3) 자궁경부 세포진 검사를 통해 HPV 감염이 있는 대상이 향후 유방암 발생 위험도가 높은지를 알아보고, (4) HPV 백신 접종 여부에 따라 유방암 발생이 어떻게 차이나는가를 규명할 것을 제안한다. 이런 연구들을 통해 HPV와 유방암 위험 간의 관련성이 확보된다면, 자궁경부암 예방을 위한 HPV 백신 접종은 유방암 예방까지 이룰 수 있을 것이다 [40, 57-59]. 한국의 현실에서는 가임기 여성에게 이루어지는 풍진 예방접종때, HPV 접종을 같이 하는 전략도 검토해 볼 수 있겠다. 본 논고가 한국 여성을 대상으로 역학적 및 실험적 연구가 활성화되는데 자극이 되길 바라며 이만 줄인다.

감사의 글

본 연구는 대한암연구재단의 2013년도 연구비 지원을 받음 (No. 2013-2).

References

1. Jemal A, Bray F, Center MM, Ferlay J, Ward E, Forman D. Global cancer statistics. CA Cancer J Clin 2011;6:69-90.
2. Ferlay J, Shin HR, Bray F, Forman D, Mathers C, Parkin DM. Estimates of worldwide burden of cancer in 2008: GLOBOCAN 2008. Int J Cancer 2010;127:2893-917.
3. Shin HR, Joubert C, Boniol M, Hery C, Ahn SH, Won YJ, et al. Recent trends and patterns in breast cancer incidence among Eastern and Southeastern Asian women. Cancer Causes Control 2010;21:1777-85.
4. Keramatinia A, Mousavi-Jarrahi SH, Hiteh M, Mosavi-Jarrahi A. Trends in incidence of breast cancer among women under 40 in Asia. Asian Pac J Cancer Prev 2014;15:1387-90.
5. Jung KW, Won YJ, Kong HJ, Oh CM, Lee DH, Lee JS. Cancer statistics in Korea: incidence, mortality, survival, and prevalence in 2011. Cancer Res Treat 2014;46:109-23.
6. Moore MA. Cancer control programs in East Asia: evidence from the international literature. J Prev Med Public Health 2014;47:183-200.
7. Bray F, McCarron P, Parkin DM. The changing global patterns of female breast cancer incidence and mortality. Breast Cancer Res 2004;6:229-39.
8. Youlden DR, Cramb SM, Dunn NAM, Muller JM, Pyke CM, Baade PD. The descriptive epidemiology of female breast cancer: an international comparison of screening, incidence, survival and mortality. Cancer Epidemiol 2012;36:237-48.
9. Shen YC, Chang CJ, Hsu C, Cheng CC, Chiu CF, Cheng AL. Significant difference in the trends of female breast cancer incidence between Taiwanese and Caucasian Americans: Implications from age-period-cohort analysis. Cancer Epidemiol Biomarkers Prev 2005;14:1986-90.
10. Bae JM. On the benefits and harms of mammography for breast cancer screening in Korean women. Korean J Fam Pract 2014;4:1-6. (Korean)
11. Lee JH, Yim SH, Won YJ, Jung KW, Son BH, Lee HD, et al. Population-based breast cancer statistics in Korea during 1993-2002: incidence, mortality, and survival. J Korean Med Sci 2007;22(Suppl):S11-6.
12. Gardner MJ, Osmond C. Interpretation of disease time trends: is cancer on the increase? A simple cohort technique and its relationship to more advanced models. J Epidemiol Commy Health 1983; 37: 274-8.
13. Kim EH, Bae JM. A potential availability of dense breast for prevention of breast cancer in Korean women. J Fam Pract 2004. (in press) (Korean)
14. Boyd NF, Lockwood GA, Byng JW. Mammographic densities and breast cancer risk. Cancer Epidemiol Biomarkers Prev 1998;7:1133-44.
15. Boyd NF, Guo H, Martin LJ, Sun L, Stone J, Fishel E, et al. Mammographic density and the risk and detection of breast cancer. N Engl J Med 2007;356:227-36.

16. Boyd NF, Rommens JM, Vogt K, Lee V, Hopper JL, Yaffe M, et al. Mammographic breast density as an intermediate phenotype for breast cancer. Lancet Oncol 2005;6:798-808.

17. Veronsei U, Boyle P, Goldhirsch A, Orecchia R, Viale G. Breast Cancer. Lancet 2005;365:1727-41.

18. Bertrand KA, Tamimi RM, Scott CG, Jensen MR, Pankratz VS, Visscher D, et al. Mammographic density and risk of breast cancer by age and tumor characteristics. Breast Cancer Res 2013;15:R104.

19. Cho JJ, Song HJ, Koh EY, Song YM, Han BK, Yun YS, et al. Mammographic breast density and risk factors of breast cancer in Korean women using multicenter study. J Korean Acad Fam Med 2006;27:33-41.

20. Kang EY, Shin JH, Kang SG, Hwang YN, Cha ES, Song SW. Relationship between mammographic dense breast and other risk factors of breast cancer in Korean women. J Korean Acad Fam Med 2007;28:937-42.

21. Park IH, Ko KR, Joo JN, Park BR, Jung SY, Lee SY, et al. High volumetric breast density predicts risk for breast cancer in postmenopausal, but not premenopausal, Korean women. Ann Surg Oncol. Epub 2014 Jun 17. [PMID: 24934582]

22. Kim SH, Kim MH, Oh KK. Analysis and comparison of breast density according to age on mammogram between Korean and Western women. J Korean Radiol Soc 2000;42:1009-14.

23. El-Bastawissi AY, White E, Mandelson MT, Taplin S. Variation in mammographic breast density by race. Ann Epidemiol 2001;11:257-63.

24. Yaghjyan L, Colditz GA, Rosner B, Tamini RM. Mammographic breast density and subsequent risk of breast cancer in postmenopausal women according to the time since the mammogram. Cancer Epidemiol Biomarkers Prev 2013;22:1110-7.

25. Bae JM, Shin SY, Kim EH, Kim YN, Nam Cm. Distribution of dense breasts using screening mammography in Korean women: a retrospective observational study. Cancer Res Treat 2014. (in press)

26. Lawson JS, Glenn WK, Whitaker NJ. Breast cancer as an infectious disease. Women Health 2010;6:5-8.

27. Lawson JS, Kan CY, Iacopetta BJ, Whitaker NJ. Are some breast cancers sexually transmitted? Br J Cancer 2006;95:1708.

28. Amarante MK, Watanabe MAE. The possible involvement of virus in breast cancer. J Cancer Res Clin Oncol 2009;135:329-37.

29. Mesa-Tejada R, Keydar I, Ramanarayanan M, Ohno T, Fenoglio C, Spegelman S. Detection in human breast carcinomas of an antigen immunologically related to a group-specific antigen of mouse mammary tumor virus. Proc Natl Acad Sci USA 1978;75:1529-33.

30. Labat ML. Possible retroviral etiology of human breast cancer. Biomed Pharmacother 1998;52:6-12.

31. Glenn WK, Heng B, Delprado W, Iacopetta B, Whitaker NJ, Lawson JS. Epstein-Barr virus, human papillomavirus and mouse mammary tumour virus as multiple viruses in breast cancer. PLoS ONE 2012;7:e48788.

32. Orem J, Mbidde EK, Lambert B, de Sanjose S, Weiderpass E. Burkitt's lymphoma in Africa, a review of the epidemiology and etiology. African Health Sci 2007;7:166-75.

33. Labrecque LG, Barnes DM, Fentiman IS, Griffin BE. Epstein-Barr virus in epithelial cell tumors: a breast cancer study. Cancer Res 1995;55:39-45.

34. Chu JS, Chen CC, Chang KJ. In situ detection of Epstein-Barr virus in breast cancer. Cancer Lett 1998;124:53-7.

35. Chu PG, Chang KL, Chen YY, Chen WG, Weiss LM. No significant association of Epstein-Barr virus infection with invasive breast carcinoma. Am J Pathol 2001;159:571-8.

36. Speck P, Callen DF, Longnecker R. Absence of the Epstein-Barr virus genome in breast cancer-derived cell lines. J Natl Cancer Inst 2003;95:1253-4.

37. Glaser SL, Hsu JL, Gulley ML. Epstein-Barr virus and breast cancer: state of the evidence for viral carcinogenesis. Cancer Epidemiol Biomakers Prev 2004;13:688-97.

38. Lawson JS, Günzburg WH, Whitaker NJ. Viruses and human breast cancer. Future Microbiol 2006;1:33-51.

39. Cox JT. The development of cervical cancer and its precursors: what is the role of human papillomavirus infection? Curr Opin Obstet Gynecol 2006;18(Suppl):S5-13.

40. Cuzick J. Long-term follow-up in cancer prevention trials (It ain't over 'til it's over). Cancer Prev Res 2010;3:689-91.

41. Damin APS, Karam R, Zettler CG, Claeffi M, Alexandre COP. Evidence for an association of human papillomavirus and breast carcinomas. Breast Cancer Res Treat 2004;84:131-7.

42. Wang T, Chang P, Wang L, Yao Q, Guo W, Chen J, et al. The role of human papillomavirus infection in breast cancer. Med Oncol 2012;29:48-55.

43. Simões PW, Medeiros LR, Pires PDS, Edelweiss MI, Rosa DD, Silva FR, et al. Prevalence of human papillomavirus in breast cancer. A systematic review. Int J Gynecol Cancer 2012;22:343-7.

44. Li N, Bi X, Zhang Y, Zhao P, Zheng T, Dai M. Human papillomavirus infection and sporadic breast carcinoma risk: a meta-analysis. Breast Cancer Res Treat 2011;126:515-20.

45. Choi YL, Cho EY, Kim JH, Nam SJ, Oh YL, Song SY, et al. Detection of human papillomavirus DNA by DNA chip in breast carcinomas of Korean women. Tumor Biol 2007;28:327-32.

46. Widschwendter A, Brunhuber T, Wiedemair A, Mueller-Holzner E, Marth C. Detection of human papillomavirus DNA in breast cancer of patients with cervical cancer history. J Clin Virol 2004;31:292-7.

47. Dunne EF, Unger ER, Stenberg M, McQuillan G, Swan DC, Patel SS, et al. prevalence of HPV infection among females in the United States. JAMA 2007;297:813-9.

48. Polyak K. On the birth of breast cancer. Biochemica et Biophysica Acta 2001;1552:1-13.

49. Hennig EM, Suo Z, Thoresen S, Holm R, Kvinnsland S, Nesland JM. Human papillomavirus 16 in breast cancer of women treated for high grade cervical intraepithelial neoplasia (CIN III). Breast Cancer Res Treat 1999;53:121-35.

50. Sigaroodi A, Nadji SA, Naghshvar F, Nategh R, Emami H, Velayati AA. Human papillomavirus is associated

with breast cancer in the north part of Iran. ScientificWorldJournal 2012;2012:837191.

51. Yu Y, Morimoto T, Sasa M, Okazaki K, Harada Y, Fujiwara T, et al. HPP33 DNA in premalignant and malignant breast lesions in Chinese and Japanese populations. Anticancer Res 1999;19:5057-62.

52. Tsai JH, Tsai CH, Cheng MH, Lin SJ, Xu FL, Yang CC. Association of viral factors with non-familial breast cancer in Taiwan by comparison with non-cancerous, fibroadenoma, and thyroid tumor tissues. J Med Virol 2005;75:276-81.

53. Khan NA, Casillo A, Koriyama C, Kijima Y, Umekita Y, Ohi Y, et al. Human papillomavirus detected in female breast carcinomas in Japan. Br J Cancer 2008;99:408-14.

54. Hedau S, Kumar U, Hussain S, Shukla S, Pande S, Jain N, et al. Breast cancer and human papillomavirus infection: no evidence of HPV etiology of breast cancer in Indian women. BMC Cancer 2011;11:27.

55. Antonsson A, Spurr TP, Chen AC, Francis GD, McMillan NAJ, Saunders NA, et al. High prevalence of human papillomaviruses in fresh frozen breast cancer samples. J Med Virol 2011;83:2157-63.

56. Wang FL, Chen F, Yin H, Xu N, Wu XX, Ma JJ, et al. Effects of age, breast density and volume on breast cancer diagnosis: a retrospective comparison of sensitivity of mammography and ultrasonography in China's rural areas. Asian Pacific J Cancer Prev 2013;14:2277-82.

57. Rambout L, Hopkins L, Hutton B, Fergusson D. Prophylactic vaccination against human papillomavirus infection and diseses in women: a systematic review of randomized controlled trials. CMAJ 2007;177:469-79.

58. Harputluoglu H, Dizdar Om, Altundag K. Prophylactic human papilloma virus vaccines for cervical cancer may also prevent development of breast and oropharyngeal cancers in women. Med Hypotheses 2006;67:431-2.

59. Mayeaux EJ. Harnessing the power of prevention: human papillomavirus vaccines. Curr Opin Obstet Gynecol 2006;18(Suppl):S15-21.

Table 1. Asian studies for evaluating association between human papilloma virus infection and risk of female breast cancer *

Design	Likely	Unlikely
Cancer tissues only	[51] (Japan & China, 1999)	[53] (Japan, 2008)<br>[54] (India, 2011)
Case-Control comparison	[50] (Iran, 2012)	[52] (Taiwan, 2005)

* [Reference number] (nation, the year of publication)

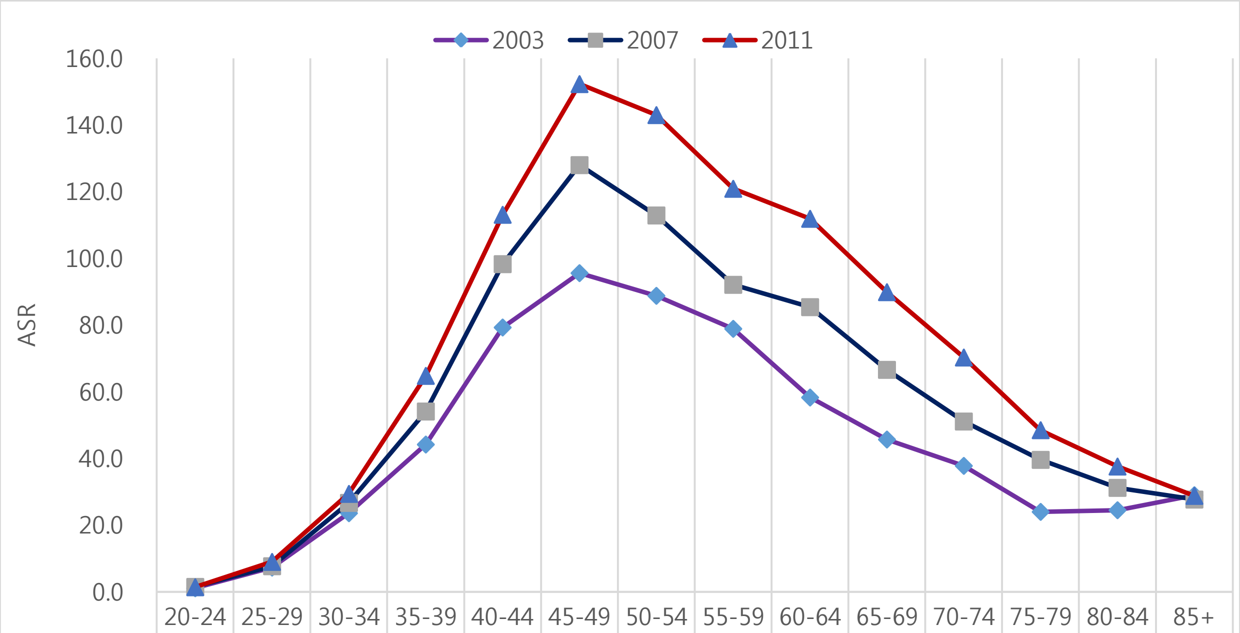

	20-24	25-29	30-34	35-39	40-44	45-49	50-54	55-59	60-64	65-69	70-74	75-79	80-84	85+
2003	1.1	7.3	23.6	44.2	79.3	95.6	88.8	78.9	58.3	45.7	37.8	24.0	24.5	29.0
2007	1.5	7.7	26.7	54.1	98.3	128.0	112.9	92.1	85.4	66.6	51.1	39.6	31.2	27.7
2011	1.4	9.0	29.4	64.8	113.1	152.3	143.0	120.9	111.9	89.9	70.3	48.5	37.6	28.8

Figure 1. Curves of incidence rates of breast cancers in Korean women by age group and 4-year interval

한글초록

유방암은 한국여성에서 갑상선암 다음으로 높은 발생률을 보이는 원발암이다. 최근 10년간 발생률은 증가하는 추세를 보이는 가운데, 연령별 발생률 곡선은 45-49세를 정점으로 단봉성을 보이는 양상이 계속 유지되고 있다. 이는 서구의 식생활습관에 따른 코호트 효과를 설명하기 어려운 현상이다. 이에 저자는 기존의 근거들을 기반하여 우리나라 여성 유방암의 위험요인으로 유방밀도와 바이러스 감염의 두 가설을 제시한다. 이 두 가설을 규명한다면, 한국여성에서의 유방암 발생에 관여하는 새로운 위험요인을 알아낼 수 있고 유방암 관리의 효과적인 대책을 수립할 수 있을 것이다.
